# Explainable machine learning models for mortality prediction in patients with sepsis in tertiary care hospital ICU in low- to middle-income countries

**DOI:** 10.1186/s40635-025-00765-5

**Published:** 2025-06-03

**Authors:** Saumya Diwan, Vinay Gandhi, Esha Baidya Kayal, Puneet Khanna, Amit Mehndiratta

**Affiliations:** 1 Centre for Biomedical Engineering, Indian Institute of Technology Delhi, Hauz Khas, New Delhi, 110016 India; 2 School of Medicine, Emory University, Atlanta, USA; 3 Department of Anaesthesiology, Pain Medicine and Critical Care, All India Institute of Medical Sciences New Delhi, New Delhi, India; 4 Department of Biomedical Engineering, All India Institute of Medical Sciences New Delhi, New Delhi, India

**Keywords:** Critical care, Explainable artificial intelligence, Machine learning, Mortality prediction, Sepsis, ICU

## Abstract

**Introduction:**

Mortality in sepsis patients remains a challenging condition due to its complex nature. It is an even more prevalent health problem in low- and middle-income countries demanding costly treatment and management. This study proposes an explainable artificial intelligence-based approach towards mortality prediction for patients with sepsis admitted to intensive care unit (ICU).

**Methods:**

A total of 500 patients (*N* = 500, male: female = 262:238, age = 45.96 ± 20.92 years) with sepsis were analyzed retrospectively. We utilize SHapley Additive exPlanations (SHAP) method to gain insights into the preliminary model’s learnings regarding the wide array of demographic, clinical, radiological, and laboratory features. The clinical insights were used for feature selection to fetch the top *t* = 80% feature spread as well as to derive empirical findings from feature dependence plots which could find application in periphery hospital settings. Four machine learning algorithms, Random Forest, XGBoost, Extra Trees and Gradient Boosting classifiers were trained for the binary classification task (discharge from ICU and death in ICU) with the selected influential feature set.

**Results:**

The Extra Trees Classifier showed the best overall performance with AUROC score: 0.87 (95% CI 0.80–0.93), Accuracy: 0.79 (95% CI 0.71–0.86), F1 score: 0.78 (95% CI 0.69–0.86), Precision: 0.88 (95% CI 0.78–0.98) and Recall: 0.70 (95% CI 0.57–0.82). All four models perform significantly well on dataset with AUROC scores ranging from 0.81 (CI 0.73–0.89) to 0.87 (CI 0.80–0.93) and F1 scores ranging 0.74 (CI 0.64–0.83) to 0.78 (CI 0.69–0.86) on the hold-out test set and were stable over fivefold cross-validation prior to testing.

**Conclusions:**

The proposed approach could provide preemptive estimations into prognostication and outcome prediction of patients with sepsis in low-resource settings. This will aid in clinical decision-making, resource allocation and research for new treatment modalities.

**Supplementary Information:**

The online version contains supplementary material available at 10.1186/s40635-025-00765-5.

## Introduction

Sepsis has a non-uniform nature and is a complex syndrome of physiologic and biochemical abnormalities. With its complex characteristics, involving organ dysfunction, it can lead to severely increased chance of patient mortality in cases of improper management of sepsis patients in hospital settings [1]. Even reliable measuring of sepsis incidence and trends is a challenging task [2]. It is estimated that in 2017, about 48·9 million sepsis incidences were recorded worldwide and around 11·0 million sepsis-related mortalities (around 19·7% of all global deaths) [3]. Due to several factors such as growth in clinical awareness, changes made in diagnostics and coding protocols and non-standardized definitions, there is an uncertainty as to the accuracy of reported trends as well as marked heterogeneity in incidence and mortality rates [2, 4–8]. This is also a significant problem in terms of the cost of treatment and management. Moreover, the mortality due to sepsis has a higher occurrence in low- and middle-income countries/low-income counties (LMICs/LICs) with respect to high-income countries (HICs) [9]. The highest burden of sepsis was noted in the Global Burden of Disease study to be in LMICs accounting for 85% of total sepsis-related deaths worldwide [10]. Therefore, preemptive estimation into the septic patient’s outcome could greatly assist clinical decision-making to prepare in terms of treatment planning, resource allocation as well as cost estimation. 

Sequential Organ Failure Assessment (SOFA) [11] and Acute Physiology and Chronic Health Evaluation II (APACHE-II) [12] are the two most common scores used to stratify sepsis patients. However, these severity scoring systems were created and validated long ago and the validity of this score primarily stems from analyzing over a million ICU electronic health records of critically ill patients with suspected sepsis in HICs. The differences in patients, pathogens, and clinical management capacity between HICs and LMICs are substantial. Consequently, it remains uncertain whether these scores are applicable across various types of infections, hospital locations, and countries [13]. Although, the conventional severity score showed fair discriminatory ability for predicting ICU mortality with a range of AUROC: 0.67–0.8; artificial intelligence (AI)-based models to predict mortality in sepsis patients showed higher discriminating abilities than the severity scores showing AUROC: 0.86–0.97 [14–16]. In a comprehensive meta-analysis over 50 studies developing a total of 104 machine learning model for predicting sepsis-related death, Zhang et al. [17] demonstrated overall C-index of 0.799 (95% CI 0.779–0.819) and 0.774 (95% CI 0.763–0.785) in the training and validation sets, respectively, for ML models, while overall C-index of the severity scoring systems was 0.717 (95% CI 0.673–0.761) and 0.689 (95% CI 0.633–0.745), respectively.

Therefore, there is an inherent need to create appropriate accountable data-driven approaches in emergency healthcare. The goal of such studies is to eventually serve as AI-based clinical decision support systems with the clinical community being the interactive user. However, adoption and credibility of such tools get severely hampered by reasons such as its black-box nature and lack of domain knowledge as well as interpretability [18]. Explainability plays a significant role in terms of technological, legal, medical, and patient perspectives [19]. Moreover, this may further serve towards alignment of model performance with clinical protocols and objectives [20].

While there have been several developments with regard to studies predicting in-hospital sepsis-related mortality utilizing machine learning (ML) and deep learning (DL) algorithms [15–32], most of such contemporary studies [21–24, 26, 28–31] are based around the large Multiparameter Intelligent Monitoring in Intensive Care (MIMIC) database [33]. While being a comprehensive dataset, the data in the MIMIC database are heterogeneous and high dimensional with over 18,000 collected variables [34]. Moreover, the outcome variable: in-hospital-mortality is imbalanced (~ 1:9 ratio of deaths to survivors) which may hamper the performance of predictive models [35]. Fewer studies involved in-hospital ICU data [15, 27] and emergency department data [16, 25, 32] from HICs. Throughout the literature, to our knowledge, we observed a lack of studies specifically catering or extending application to LMIC environments.

Feature selection is another very critical area which becomes essential due to high dimensional healthcare datasets and associated variability in the features [36]. Feature selection essentially reduces dimensionality of the input data and can improve model performance by multiple ways such as decreasing the learning speed, reducing model complexity or by increasing generalization capacity and classification accuracy [37]. Selection of suitable representative features can significantly reduce computational costs and lead to greater understanding of the problem. Another observed shortcoming was, there seem to be vary less studies [15, 22] dedicated to interpretability and explainability of models used for feature selection as well as integration of clinical insights.

With regard to the aforementioned, this research investigates a novel machine learning model-based prediction of mortality in patients with sepsis in ICU settings. A twofold utilization of explainable AI study was employed: firstly, feature engineering and selection and secondly, for generating data-driven insights into the model’s learnings using SHapley Additive exPlanations (SHAP) [38]. Finally using the selected features, a comparative set of ML models were trained to predict mortality through a binary classification task.

## Materials and methods

### Dataset

The study was approved by the Institute Ethic Committee of the All India Institute of Medical Sciences (AIIMS), New Delhi, India, with ethical approval number, IECPG-190/20.04.2023, RT-05/07.06.2023. A retrospective dataset of septicemia patients, admitted to different Intensive Care Units (ICUs) in AIIMS New Delhi, India, from January 2021 to January 2024, was collected from ICU clinical records. The inclusion criteria were patients with more than 18 years of age and diagnosed with sepsis (according to Sepsis-3 definition [39]) during admission to ICU. The exclusion criteria were patients with non-septic shock and already developed refractory septic shock during ICU admission. The outcome of the patient, i.e., mortality or discharge (discharge from ICU) was taken as the target variable (in binary variable format). A total of 500 patients (*N* = 500, male: female = 262:238, age = 45.964 ± 20.921 years) were randomly included in the study taking 250 patients each in the mortality and discharge group to match the mortality–discharge ratio 1:1. Clinical data of patients were collected only once within the first 24 h of admission to the ICU and directly entered into an Excel sheet along with the demographic data for further analysis. The dataset contained total 108 parameters comprising patient demographics, clinical, ventilatory, blood-gas, laboratory, respiratory and cardiovascular parameters; information about source of sepsis, organ dysfunction, metabolic disorders; interventions, regarding oxygenation, vasopressors, antibiotics and some miscellaneous parameters like duration of illness to ICU stay, etc. Details of different parameters are presented in Supplementary Table 1. Patient demography and salient clinical parameters in mortality and discharge groups are presented in Table 1.

**Table 1:** Patient characteristics and baseline clinical and laboratory parameters

Parameters	Mortality group (*n* = 250)	Discharge group (*n* = 250)	*p*-value
Demographic parameters			
Age	46.5 [7–93]	40.5 [16–100]	0.334
Sex—female	102 (40.8)	136 (54.4)	0.003
Weight	65 [20–110]	65 [33–170]	0.533
No substance abuse	161 (64.4)	192 (76.8)	0.003
Smoking	73 (29.2)	41 (16.4)	0.001
Alcoholism	63 (25.2)	40 (16)	0.015
Other substance abuse	26 (10.4)	11 (4.4)	0.017
Duration of illness before ICU admission	13 [1–365]	7 [1–120]	< 10^–3^
Clinical parameters			
Glasgow Coma Scale (GCS)	9 [3–15]	11 [3–15]	< 10^–3^
Heart rate	117 [45–183]	100 [48–180]	< 10^–3^
Blood pressure systolic	110 [60–190]	121 [11–200]	< 10^–3^
Blood pressure diastolic	64 [30–120]	72 [40–110]	< 10^–3^
Respiratory rate	25 [12–40]	24 [12–732]	0.851
Saturation of peripheral oxygen (SpO2)	98 [66–1010]	99 [84–100]	0.527
Intubated	198 (79.2)	129 (51.6)	< 10^–3^
Lactate (mmol/L)	2.5 [0.11–19.3]	1.2 [0.24–9.4]	< 10^–3^
pH	7.32 [6.7–8]	7.39 [6.92–7.57]	< 10^–3^
PaO2/FiO2-ratio	193 [44–492]	232.15 [44.8–495.8]	0.026
Hb (g/dl)	8.8 [3.6–12.12]	9.6 [4.5–11.5]	0.463
TLC (/mm^3^)	12,920 [0.71–116600]	12,140 [9.62–85550]	0.993
PLT (/mm^3^)	1.03 [0.07–70]	1.6 [0.07–77]	0.669
PT (s)	17.75 [10.2–41.7]	14.2 [8.8–34.9]	< 10^–3^
INR	1.54 [0.8–3.8]	1.2 [0.45–2.6]	0.001
APTT (s)	41.61 [17.8–88]	33.4 [1.52–75]	0.008
Urea (mg/dl)	72.5 [2–318]	48.5 [7–326]	< 10^–3^
Creatinine (mg/dl)	1.8 [0.3–12.2]	1 [0.2–13]	0.064
Albumin (g/dl)	2.45 [1–4.7]	2.8 [1.4–5.6]	< 10^–3^
SOFA score	10 [1–20]	6 [2–16]	< 10^–3^
Source of sepsis			
Pulmonary	169 (67.60)	159 (63.6)	0.397
Intra-abdominal	48 (19.2)	36 (14.4)	0.188
Genito-urinary	25 (10)	36 (14.4)	0.172
Surgical site infection (SSI)	31 (12.4)	21 (8.4)	0.187
Central nervous system (CNS)	9 (3.6)	9 (3.6)	1.00
Other	42 (16.8)	55 (22)	0.175
Organ dysfunction			
Respiratory (PaO2/FiO2 ratio < 300)	189 (75.6)	166 (66.4)	0.03
Liver (INR > 1.5 and total bilirubin > 2 mg/dl)	136 (54.4)	73 (29.2)	< 10^–3^
Hemodynamic (MAP < 65 mmHg or vasopressor requirement)	186 (74.4)	96 (38.4)	< 10^–3^
Central nervous system (GCS < 15)	138 (55.2)	67 (26.8)	< 10^–3^
Renal (creatinine > 1.5 mg/dl)	155 (62)	98 (39.2)	< 10^–3^
Septic shock	186 (74.4)	91 (36.4)	< 10^–3^

Values are presented as median [range] or total count (%). The p values were calculated using Chi-squared test for categorical parameters and t test for continuous parameters

*PaO2* partial pressure of oxygen, *FiO2* fraction of inspired oxygen, *HB* hemoglobin, *TLC* total leucocyte count, *PLT* platelet count, *PT* prothrombin time, *INR* international normalized ratio, *APTT* activated partial thromboplastin time, *MAP* mean arterial pressure, *SOFA* score Sequential Organ Failure Assessment score

### Preprocessing

Data were first cleaned by removing duplicate and irrelevant observations, and structural errors to ensure uniformity of data across each clinical parameter. Textual information in terms of antibiotics and procedures invoked were converted into categorical and count-based variables by clinical inputs. The antibiotic groups were clinically engineered and categorized. The patient data acquired were clinically evaluated to remove any redundant features. The nature of data was mixed including continuous, categorical, and binary data points. One-hot encoding of categorical data points, i.e., conversion of categorical data from string or numeric values to binary values across each category was performed. To handle the missing values, features with more than 30% missing values were excluded from the dataset. For dichotomous features, missing data were replaced with mode of respective feature across the sample and for continuous valued features, Multivariate Imputer with Bayesian Ridge Estimator (https://scikit-learn.org) was used to replace the missing value in the respective feature across the sample. The features with missing values that were considered for preprocessing are listed in Supplementary Table 2. After preprocessing, a total of 138 features were used for further analysis.

### Correlation and predictive power score analysis

Pearson correlation analysis was performed on the resulting features as displayed in Supplementary Fig. 1. Similarly, predictive power scores were determined to investigate the nonlinear feature relations. It is to be noted that most features did not show any significant correlation or predictive power scores. Therefore, all 138 features were retained for further analysis [36, 37].

### Feature analysis using explainable AI

Machine learning-based models are usually considered a black-box by the clinical community. They may present efficient solutions, but do not prove much justification as to the implicit learnings from the data and are usually selected based on their performance rather than interpretability and practical insights [40]. Since we are dealing with a high dimensional dataset, SHAP [38] was utilized to provide local and global insights with regard to the model output providing the most influencing features. SHapley Additive exPlanations (SHAP) is a game theory based model interpretability approach that provides post hoc estimations of the conditional impact of a feature on the model’s prediction with respect to all other features [41]. SHAP methodology is model agnostic and can be used across different machine learning models for comparison. It essentially computes the sum of feature contributions for each instance to give an explanation of the predictive output. It determines a value (SHAP value) that signifies the role of each feature in the model's optimal outcome and ranks the features in order of importance. These values are useful for assessing the significance of individual features and its impact on the model's outcomes. Specifically, here the Tree Explainer [42] was used with the baseline estimator as XGBoost [43] that utilizes previous models’ residuals to create new models iteratively to make predictions. It has been noted to outperform even deep learning models for tabular data and does not require any significant fine-tuning for the dataset [44].

The complete dataset was split into training and hold-out test set in the ratio of 4:1. An XGBoost instance was trained on the former data subset considering as the baseline estimator for Tree SHAP explainer allowing for the exact computation of SHAP values for tree ensemble methods. Following these the local and global explanations were determined via SHAP. The purpose of this was twofold: first was the identification of most influential features to the model’s predictive outputs as well as looking into local and global inter-feature dependencies learnt by the model. Second was identifying inter-feature relations with respect to the model. SHAP interaction plots were generated for the selected important features to visualize the impact of the individual feature towards the mortality due to septic shock among patients.

### Feature selection

The mean absolute SHAP-based feature influence values were determined and plotted. The summation of these was considered the total reference value, and based on the observed spread of values, features contributing to the top ‘*t*’% (*t* = 80% in this study) of information were selected. These features were then considered for further model training and performance evaluation.

### Model development and evaluation

With the selected features from the previous step, four tree-based models namely Random Forest Classifier, XGBoost Classifier, Extra Trees Classifier and Gradient Boosting Classifier were considered for prediction model development. Prediction models using those four algorithms were also developed with the current standard of practice SOFA scores separately for comparison with the proposed model. The training dataset comprising 400 samples was used to train the four classifiers with fivefold cross-validation. The hold-out test set of 100 patients was retained separately to ensure no data leakage and to evaluate model efficacy. Performance metrics such as area under the receiver operating characteristics curve (AUROC) values, accuracy, F1 score, precision and recall were evaluated for each cross-validation fold to assess the training of the models. Mean values of all metrics across the fivefold cross-validation were evaluated for the four classifiers and compared. Finally, the performances of trained classifiers were evaluated on the hold-out test set and compared.

## Results

### Feature selection

After preprocessing, the distributions of mean |SHAP values| of the resulting influential features are shown in Supplementary Fig. 2. Clinical, laboratory and blood-gas features occupied 71% (36%, 20%, and 15%, respectively) of the total SHAP value spread. Ventilatory, intervention, demographic, dysfunction and disorder, and time duration features held the remaining 29% of the total SHAP value spread. Based on mean absolute SHAP value a subset of *n* = 39 most influential features representing 80% of total SHAP value spread were selected for further model development (Supplementary Fig. 3).

### SHAP-based feature analysis

**Fig. 1:**
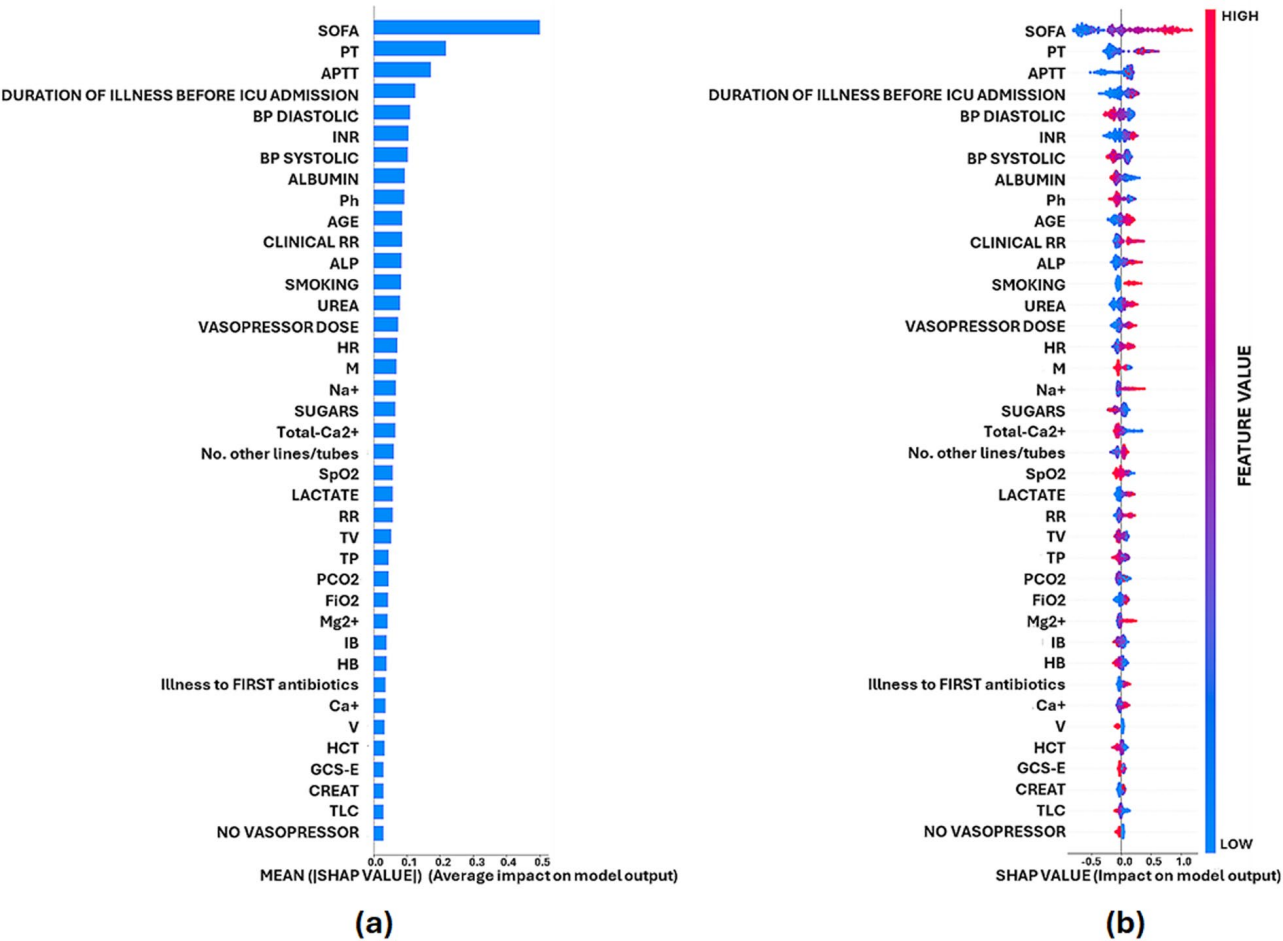
Feature-wise impact of features on model output. **a** Bar chart representing mean absolute SHAP values of features. **b** Density scatter plot showing SHAP value with directional information on model output. Values towards the + ve x-axis (right) signifies the tendency of the parameter to drive the model output towards giving a mortality prediction and -ve axis towards an alive state prediction. Red signifies a higher value of the feature and blue a lower value

Figure 1.a shows the average SHAP value magnitudes of the selected 39 most influential features in a bar chart in descending order. SOFA score, Prothrombin time (PT), Activated Partial Thromboplastin Time (APTT) were observed to be the features having a significant impact on the model output. These were followed by the duration of illness of the patient before ICU admission, International normalized ratio (INR) as well as the systolic and diastolic blood pressure (BP).

A density scattered summary plot of SHAP values is shown in Fig. 1b, wherein, each point depicts a single sample, i.e., a single patient. The actual SHAP value (the logarithmic odds for predicting patient mortality) is represented along the X-axis where a higher SHAP value indicates a higher chance of mortality in comparison to a lower SHAP value. Similar to Fig. 1a, along the Y-axis, features are arranged based on their importance, which is given by the means of their absolute Shapley values. The color scheme represents the features’ value. It can be noted that SOFA score affects a large number of predictions by a significant amount. PT follows closely after it with a similar nature. However, APTT, PT, duration of illness, INR and BP seem to affect most predictions but by a small amount.

**Fig. 2:**
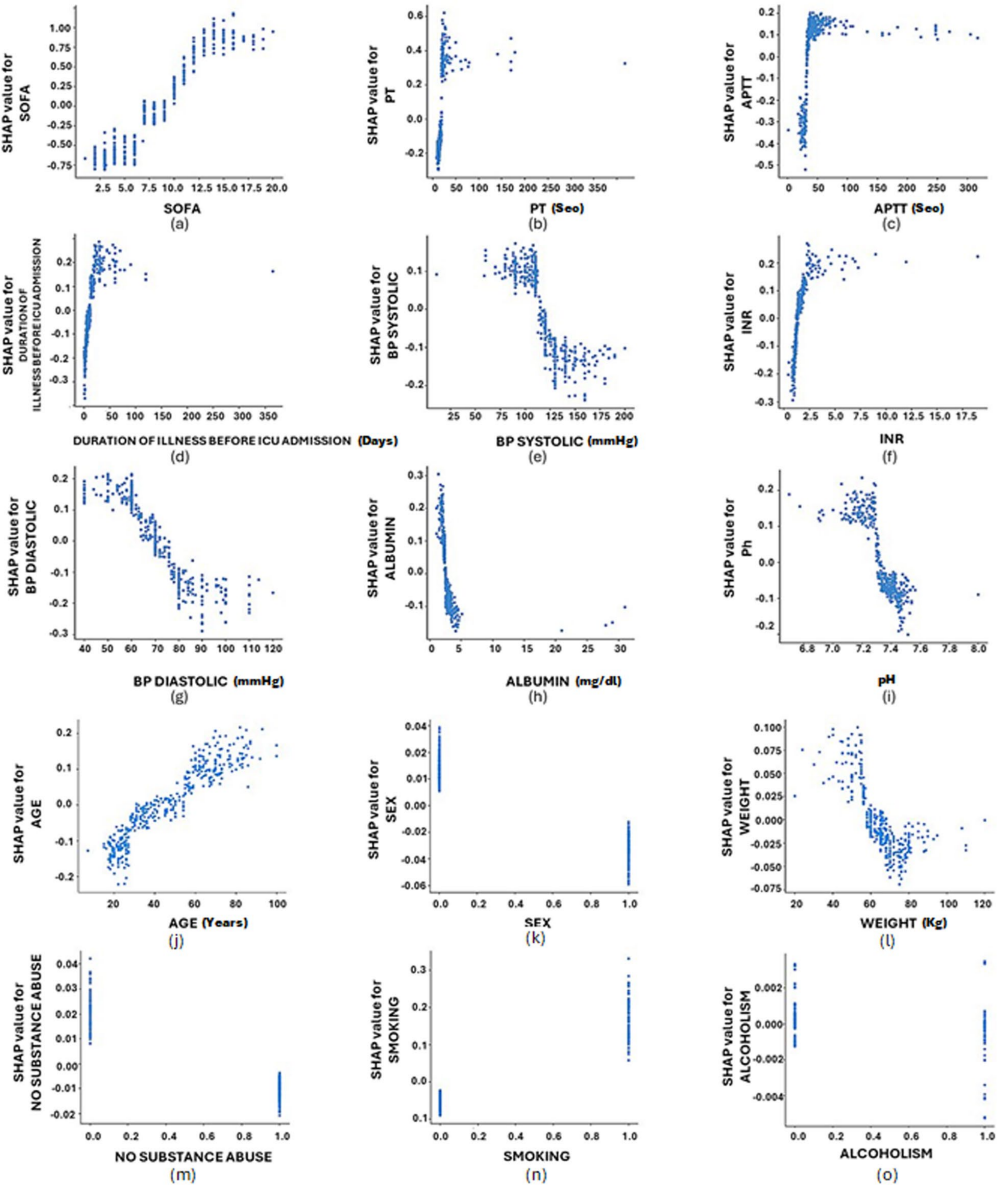
SHAP-based dependence plots of most influential clinical features such as **a** SOFA scores, **b** prothrombin time (PT), **c** activated partial thromboplastin time (APTT), **d** duration of illness before ICU admission, **e** blood pressure (BP) systolic, **f** international normalized ratio (INR), g blood pressure (BP) diastolic, **h** albumin and **i** pH and demographic features such as **j** age, **k** sex, **l** weight, **m** no substance abuse, **n** smoking, and **o** alcoholism. Each point in the plot corresponds to an individual patient and the position of a said point along the x-axis corresponds to the value of the feature and along y-axis corresponds to the SHAP value for the feature under consideration. Please note that the scale of the y-axis is not the same for all plots; this was set to be able to capture the effects of all top features

Figure 2 depicts individual dependence plots for the top nine features from SHAP analysis and patients’ demographic features, showing the effect of a single feature on the overall models’ prediction performance. Some notable observations can be made from the dependency plots regarding the nonlinear effects of the features on the model output. For the SOFA score, a turning point was observed where patients with scores lower than 10 seem to be at a lower mortality risk, however, past this score the risk was found to increase steeply (Fig. 2a). Increased INR of patients was found to show a sharp push towards mortality (Fig. 2f) while, lower systolic and diastolic BP (< 120 mmHg and < 70 mmHg, respectively) show an increased risk of mortality (Fig. 2e and g). Albumin levels below ~ 2.5 mg/dl and pH under ~ 7.35 also showed a sharply increased risk for mortality among ICU patients (Fig. 2h and i). Concerning patient demographics, patients with more than 50 years of age and above 60 kg of weight were observed to be at a higher risk of mortality and the risk was found to increase with increasing age and weight (Fig. 2j and l, respectively). In our dataset, we also observed that females being at a lower risk than males (Fig. 2k) and non-smoking patients with no substance abuse and no alcoholism were at a lower risk than those partaking in such activities (Fig. 2m–o).

### Predictive model performance

**Fig. 3:**
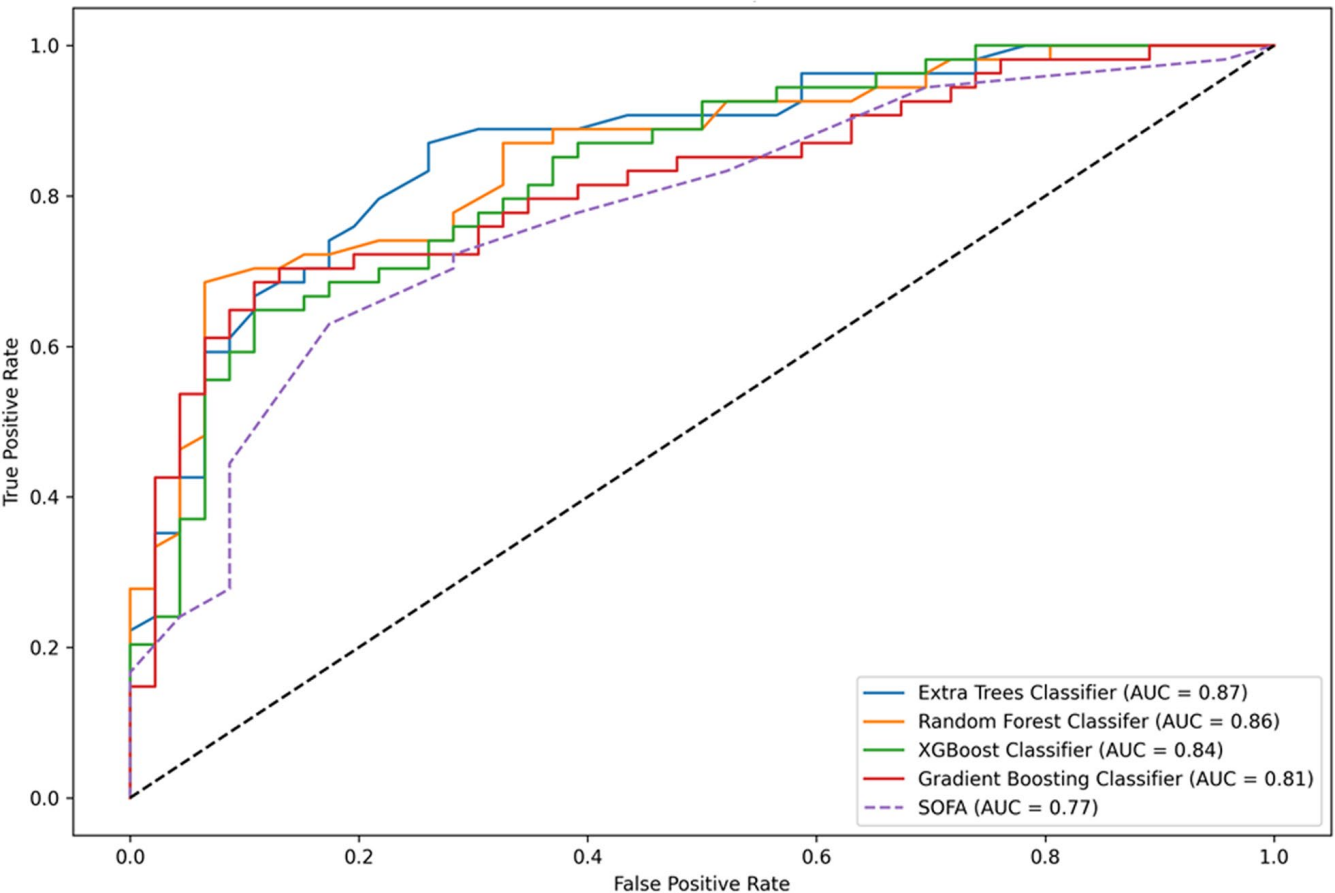
Plots show AUROC curves for model testing on the hold-out test dataset to compare the performance using SHAP-based selected feature set with Extra Trees Classifier, Random Forest Classifier, XGBoost Classifier, and Gradient Boosting Classifier and model using only SOFA score as a feature

The performance of the prediction models using selected feature set and SOFA score alone is summarized in Table 2. Using SOFA scores alone, all four models demonstrated average cross-validation AUROC in the range 0.786 ± 0.041 to 0.792 ± 0.043 and AUROC of 0.77 (95% CI 0.67–0.86) on the hold-out test dataset. While all the proposed ML models using the selected feature set produced superior accuracies on our dataset showing average cross-validation AUROC ranging from 0.822 ± 0.015 to 0.843 ± 0.018 and F1 scores ranging 0.726 ± 0.025 to 0.779 ± 0.042 for training data and on hold-out test dataset showed AUROC ranging from 0.81 (95% CI 0.73–0.89) to 0.87 (95% CI 0.80–0.93) and F1 scores ranging 0.74 (95% CI 0.64–0.83) to 0.78 (95% CI 0.69–0.86). ROC analysis for four models using the selected feature set demonstrated stable results for model training across all cross-validation folds (Supplementary Fig. 4). Figure 3 shows the comparative ROC analysis for testing the four classification models on the hold-out test dataset using the selected feature set and the model using SOFA scores alone. Extra Trees Classifier showed the best overall testing performance with AUROC: 0.87 (95% CI 0.80–0.93), accuracy: 0.79 (95% CI 0.71–0.86), F1 score: 0.78 (95% CI 0.69–0.86), precision: 0.88 (95% CI 0.78–0.98) and recall: 0.70 (95% CI 0.57–0.82). While using SOFA score alone, the model showed a comparatively lower testing AUROC of 0.77.

**Table 2:** Comparative modeling analysis for selected features

ML models	Feature used		Fivefold cross-validation					Hold-out test				
			AUROC	Accuracy	F1 score	Precision	Recall	AUROC	Accuracy	F1 score	Precision	Recall
Random Forest Classifier	SHAP-based features		0.832 ± 0.019	0.758 ± 0.034	0.757 ± 0.039	0.744 ± 0.032	0.745 ± 0.054	0.86 (95% CI 0.79–0.93)	0.79 (95% CI 0.70–0.87)	0.78 (95% CI 0.68–0.86)	0.90 (95% CI 0.80–0.98)	0.69 (95% CI 0.55–0.81)
	SOFA score alone		0.786 ± 0.041	0.720 ± 0.042	0.723 ± 0.049	0.716 ± 0.067	0.734 ± 0.094	0.77 (95% CI 0.66–0.86)	0.68 (95% CI 0.59–0.77)	0.67 (95% CI 0.56–0.77)	0.75 (95% CI 0.62–0.87)	0.61 (95% CI 0.48–0.73)
XGBoost Classifier	SHAP-based features		0.843 ± 0.018	0.785 ± 0.038	0.779 ± 0.042	0.785 ± 0.043	0.775 ± 0.064	0.84 (95% CI 0.76–0.91)	0.75 (95% CI 0.66–0.83)	0.75 (95% CI 0.65–0.84)	0.82 (95% CI 0.70–0.93)	0.69 (95% CI 0.56–0.80)
	SOFA score alone		0.792 ± 0.043	0.720 ± 0.042	0.718 ± 0.040	0.716 ± 0.067	0.734 ± 0.094	0.77 (95% CI 0.68–0.85)	0.68 (95% CI 0.58–0.77)	0.67 (95% CI 0.56–0.77)	0.75 (95% CI 0.62–0.87)	0.61 (95% CI 0.48–0.74)
Extra Trees Classifier	SHAP-based features		0.841 ± 0.016	0.738 ± 0.042	0.726 ± 0.025	0.777 ± 0.035	0.709 ± 0.046	0.87 (95% CI 0.80–0.93)	0.79 (95% CI 0.71–0.86)	0.78 (95% CI 0.69–0.86)	0.88 (95% CI 0.78–0.98)	0.70 (95% CI 0.57–0.82)
	SOFA score alone		0.791 ± 0.043	0.727 ± 0.051	0.723 ± 0.049	0.727 ± 0.069	0.729 ± 0.085	0.77 (95% CI 0.67–0.86)	0.68 (95% CI 0.58–0.77)	0.67 (95% CI 0.56–0.77)	0.75 (95% CI 0.61–0.87)	0.61 (95% CI 0.48–0.74)
Gradient Boosting Classifier	SHAP-based features		0.822 ± 0.015	0.750 ± 0.021	0.750 ± 0.014	0.735 ± 0.035	0.735 ± 0.035	0.81 (95% CI 0.73–0.89)	0.76 (95% CI 0.67–0.85)	0.74 (95% CI 0.64–0.83)	0.88 (95% CI 0.76–0.97)	0.65 (95% CI 0.52–0.77)
	SOFA score alone		0.791 ± 0.044	0.720 ± 0.042	0.718 ± 0.040	0.716 ± 0.067	0.734 ± 0.094	0.77 (95% CI 0.67–0.85)	0.68 (95% CI 0.59–0.77)	0.67 (95% CI 0.56–0.77)	0.75 (95% CI 0.62–0.87)	0.61 (95% CI 0.49–0.74)

Table shows mean ± standard deviation of results of fivefold cross-validation for selected features and model performance on hold-out test set with 95% confidence Intervals

## Discussion

The current study was performed to predict in-hospital mortality by using an explainable ML model in patients with sepsis admitted to the ICU. The study utilized demographic, clinical, radiological, and laboratory data of the patients admitted to the ICU for model development and validation. Even though the literature already has multiple machine learning models to predict mortality in sepsis [17], a relatively smaller number of models were developed in LMICs and LICs wherein as the demographic and clinical characteristics of patients are different from those in HICs. Therefore, the current study is of vital importance to capture the realistic and reproducible scenario in the subcontinent population. In this study all four ML models performed reasonably better (AUROC: 0.81–0.87) on the current dataset for predicting sepsis-related mortality than the current standard of practice using the severity scores SOFA alone (AUCOR: 0.77); while proposed Extra Trees Classifier exhibited the best overall performance with AUROC of 0.87.

Numerous studies in past have focused on prediction of in-hospital sepsis-related mortality showcasing a growing utility of different machine learning (ML) and deep learning (DL) algorithms, where RF and XGBoost were reported as the most preferable models [17]. In the developmental work for morality prediction models in sepsis among several ML algorithms XGBoost model has shown the best performance as reported by Bao et al. (AUROC:0.96) [21], Lu et al. (AUROC: 0.842) [22], and Choi et al. (AUROC: 0.977) [15]. On the other hand, among different ML models for mortality prediction, RF model also reported to produce the highest performance by Poucke et al. (AUROC: 0.74) [23], Kong et al. (AUROC:0.845) [24] in ICU patients and by Greco et al. [16], Taylor et al. [25] in emergency department with sepsis patients (AUROC: 0.86). Zhang et al. reported, LASSO score as the best discrimination with AUROC of 0.772 for in-hospital mortality in severe sepsis [26]. Choi et al. reported using MLP an AUROC of 0.835 for predicting mortality in sepsis [27]. A novel Quotient Basis Kernel for SVM was developed for mortality prediction in sepsis using soft-margin SVM and showed ~ 80% prediction accuracy [28]. Shin et al. leveraged word representations and embeddings out of clinical notes and along with clinical data for early prediction of mortality and showed the best AUROC of 0.85 [29]. Additionally, Shukla et al. proposed a blockchain-based clinical decision support system for similar prediction and reported AUROC of 0.79 [30]. DL-based prediction models for sepsis-related mortality were developed by Yong et al. using deep neural network and graph convolutional network [31] and by Cheng et al. [32] using CNNs, LSTM and demonstrated an improved results producing than the ML-based prediction models. In comparison to the reported ML-based models for mortality prediction in sepsis, proposed ML models have a higher predictive accuracy than the models developed by [26–28, 30, 31]. Proposed RF model (AUROC: 0.86) demonstrated a better performance on the in-hospital dataset than reported by Poucke et al. (AUROC: 0.74) [23] and Kong et al. (AUROC:0.845) [24] in ICU patients; however, was comparable with RF models as reported by Greco et al. [16], Taylor et al. [25] and Cheng et al. [32] in Emergency department with sepsis patients (AUROC: 0.86). Proposed XGBoost model (AUROC: 0.84) showed a comparable accuracy with [22]; however, had less predictive accuracy than as reported by Bao et al. (AUROC: 0.96) [21] and Choi et al. (AUROC: 0.977) [15]. This may be due to variability in the feature set included in this study while Bao et al. used MIMIC-IV data containing only electronic medical records on patients in the United States [21] and Choi et al. used only limited clinical parameters [15]. In our study, Extra Trees Classifier demonstrated a higher accuracy than the widely used RF and XGBoost models because using the entire dataset during the model training Extra Trees allows to reduce the bias in the model compared to the RF and XGBoost models.

Overall ICU mortality in our study was 41.5% and discharge rate (Discharged to ward) was 58.5%. Mortality in this study population was lower than the ICU mortality of 56.1% reported by Chatterjee et al. [45], and slightly higher to the ICU mortality of 36.7% in a multicenter study of 150 ICUs from 16 Asian countries [46]. We divided the 500 patients into two groups based on binary outcomes (discharge to the ward and death in the ICU). No significant difference was observed between the two groups in the demographic parameters like, age and weight (*p* = 0.3344 and 0.5327). However, number of male patients (*p* = 0.003), patients with more substance abuse (*p* = 0.003), smoking (*p* = 0.001), and alcoholism (*p* = 0.015) were significantly higher in the mortality group than the discharge group similar to the previous studies [47–49]. In the current study, SOFA score, PT, and APTT were found to be the highest influential parameters followed by the duration of illness before ICU admission, systolic and diastolic blood pressure at the time of ICU admission to predict mortality among ICU patients. Similar observations were reported in [22] using SHAP values for feature importance. After SHAP analysis, while the SOFA score was observed to capture the highest contributing feature, however, its two sub-components, the absolute value of bilirubin and creatinine, were also found to be informative and contributed to the model performance. Though multicollinearity among SOFA and its sub-components may not be completely ruled out, yet it is not easy to determine because of the high non-linearity of the sub-scoring systems and the range of individual components contributing to determine a single consolidated and discrete SOFA score. Further, causal relations of the demographic and clinical parameters with risk of mortality among septicemia patients were observed similar as the earlier study by Zhang et al. [50]. A higher value of SOFA, PT, and APTT were found to be associated with a higher risk of mortality, while a lower systolic and diastolic blood pressure were associated with an increasing risk of mortality in patients with sepsis. Among demographic parameters, higher age and weight were observed to be associated with a high risk of mortality, while no substance abuse, no smoking, and non-alcoholism were associated with a lower risk of mortality and are also reported earlier [47–49]. 'AGE’ was found to be an important feature after SHAP analysis, however at a lower rank compared to SOFA scores that hold a very high SHAP importance for predicting mortality due to sepsis. However, after removing SOFA scores from the feature list, ‘AGE’ showed high SHAP importance, although overall accuracy of the model reduced as SOFA was observed to be an essential feature for model functioning.

In the case of ICU patients, a wide array of data parameters is generated due to continuous monitoring. In such cases, making accurate decisions gets difficult for the clinician and, to an extent, for the machine learning algorithms, owing to their very high dimensionality. The proposed explainable feature selection methodology led us to reduce our dataset of 138 patient parameters to a key 39 parameters, representing 80% of the total spread of influence on the model output while retaining high model performance. The added data insights from such explainable predictive models in healthcare settings can help increase the trust of clinicians and drastically improve the adoption of AI-enhanced clinical decision support systems, putting them in a position of proactive care rather than reactive care delivery. This could in turn improve patient outcomes, quality of care, streamline treatment protocols, help allocate resources in an efficient manner, identify high-risk patients, develop new treatment modalities, and evaluate their efficacy [51]. This also paves the way for future modeling efforts by giving insight into what the model actually learns from the data. With the help of such approaches, unexplored patterns not so visible to clinicians, as well as traditional statistical approaches, can be unearthed. In summary, the novel contributions of this study are twofold explainable AI-based investigation into Intensive Care Unit patients’ data as well as data modeling using SHAP explanations. This encompassed identification of most influential features to the model’s predictive outputs as well as looking into local and global inter-feature dependencies learnt by the model. Further, a robust machine learning-based classification model approach for high dimensional clinical parameters of mixed data type. Finally, development of predictive models to provide preemptive estimations into prognostication and outcome prediction of patients with sepsis in low-resource settings.

There are few limitations in this study. While this study displays a promising role for explainable AI-based approaches, especially in low-resource settings, generalizability is still a challenge. The dataset in the current study is limited as compared to those in the literature, such as the MIMIC dataset [33]. To facilitate adoption in clinical settings, large-scale multi-centric model validations as well as prospective studies are required while monitoring longitudinal effects. The proposed ML model is shared as an open-source tool through GitHub (https://github.com/amitvmehndiratta/eML_Sepsis_Prediction_ICME2025) and thus it can be accessible to larger community for independent evaluation. Other sources of clinical information in the form of text, image, waveform, etc., could not be included in the proposed prediction model due to limited resources and non-availability of EMR that can be assessed in retrospective studies, which might be a common case in the low-resource settings. However, more elaborated data can add value for generalizability of the model and thus must be included in future prospective studies. The current study works only with static data. Data points from time of admission to the ICU and interventions provided have been considered; however, time series data that have not been taken into account might have further improved the performance of the models [21]. The empirical insights observed via SHAP also require prospective data to note causality. A comparative analysis using SOFA score alone was performed for prediction mortality among septicemia patients, however another commonly used risk stratification score, APACHE II, could not be performed due to insufficient data and this may be taken up in the future work.

## Conclusions

This study demonstrated the SOFA score, PT, APTT, systolic and diastolic blood pressure at the time of ICU admission and the duration of illness before ICU admission were the highest influence parameters for predicting death among patients with sepsis in ICU. With the explainable feature insights proposed ML models, specifically Extra Trees Classifier model showed satisfactory performance to stratify high-risk sepsis patients and can aid in clinical decision-making, treatment planning and resource management. A further study on prospective validation of the developed model is required, to evaluate predictive performance of the model.

## Supplementary Information

Additional file 1

## Data Availability

The datasets generated and analyzed during the current study are not publicly available due institutional data confidentiality policy but are available from the corresponding author on reasonable request.
