# Supplementary material for: Explainable machine learning models for mortality prediction in patients with sepsis in tertiary care hospital ICU in low- to middle-income countries
Source: Intensive Care Med Exp. 2025 Jun 3;13:56. doi: 10.1186/s40635-025-00765-5 (PMC12133658; doi:10.1186/s40635-025-00765-5)
Supplement: Supplementary file 1 — Additional file 1 [file 40635_2025_765_MOESM1_ESM.docx]

**Supplementary Materials**

| **Supplementary Table 1:** Broad Parameters monitored for each patient in Intensive Care Unit after preprocessing (N=138). | | | | |
| --- | --- | --- | --- | --- |
| **Data Categories** | **Features (N=138)** | | | |
| DEMOGRAPHIC PARAMETERS | AGE, SEX, WEIGHT, NO SUBSTANCE ABUSE, SMOKING, ALCOHOLISM, OTHER SUBSTANCE ABUSE | | | |
| CLINCAL PARAMETERS | GCS (E, V, M,), TEMP, HR, BP (SYSTOLIC & DIASTOLIC), RR, SpO2 | | | |
| MECHANICAL VENTILATION PARAMETERS | INTUBATED (YEAS/NO), TV, RR, PEEP, FiO2 | | | |
| BLOOD GAS PARAMETERS | LACTATE, pH, PCO2, PO2, HCO3, BE, SUGARS, Ca+, Mg2+, PaO2/FiO2-Ratio | | | |
| LABORATORY PARAMETERS | HB, TLC, PLT, PT, INR, APTT, UREA, CREATININ, Na+, K+, Cl-, Total Ca2+, TB, DB, IB, SGOT, SGPT, ALP, TP, ALBUMIN, PCT, HCT, Phosphate | | | |
| LUNG ULTRASOUND | LUNG ZONES, PLEURAL EFFUSION, CONSOLIDATION | | | |
| CRITICAL CARE ECHO | HEART-RA, HEART-RV, HEART-LA, HEART- LV, CONTRACTILITY-RV, CONTRACTILITY-LV, LVEF CLASS MARK, IVC SIZE, IVC VARIATION, DVT Y/N | | | |
| ORGAN DYSFUNCTION AND DISORDERS | SOFA, SOURCE OF SEPSIS, ORGAN DYSFUNCTION, METABOLIC ACID BASE DISORDERS, NUMBER OF COMORBIDITIES | | | |
| TREATMENT AND INTERVENTIONS | OXYGEN THERAPY, VASOPRESSOR DOSE, STEROIDS, ANTIBIOTICS STARTED ON DAY ONE OF ICU ADMISSION, ANTIBIOTICS TILL DAY OF ADMISSION, NUMBER OF VENUS LINES, NUMBER OF OTHER LINES/TUBES, | | | |
| TIME AND DURATIONS | DURATION OF ILLNESS BEFORE ICU ADMISSION, TYPE OF ICU ADMISSION, ILLNESS TO FIRST ANTIBIOTICS | | | |
| OUTCOME VARIABLE | DEATH(ICU), DISCHARGED TO WARD, SHIFTED TO ANOTHER ICU. | | | |
| GCS-Glasgow coma scale; TEMP- Temperature; HR - Heart rate; BP- Blood pressure systolic and diastolic; RR-Respiratory rate; SPO2-Saturation of peripheral oxygen; TV- Tidal volume; PEEP –Positive End Expiratory Pressure; FI02- Fraction of Inspired Oxygen; HB - Hemoglobin; TLC-Total leucocyte count; PLT- Platelet count; PT/INR- Prothrombin time/International normalized ratio; APTT- Activated Partial Thromboplastin Time; Na - Sodium; K+- Potassium; Cl—Chloride; Total ca2+- Total Calcium; TB- Total bilirubin; DB-Direct bilirubin; IB- Indirect Bilirubin; SGOT-Serum Glutamic-oxaloacetic transaminase; SGPT- Serum Glutamate-Pyruvate Transaminase; ALP- Alkaline Phosphatase; TP- Total protein; Alb: Albumin; PCT- Procalcitonin; HCT- Hematocrit; Heart RA- Right Atrium; RV- Right ventricle; LA- Left atrium; LV- Left ventricle; RV- Right ventricle; LVEF- Left ventricular ejection fraction; IVC size- Inferior venacava size; DVT- Deep vein thrombosis; SOFA score- Sequential Organ Failure Assessment score. | | | | |
| **Supplementary Table 2:** The parameters with missing values. Values are presented as total count (%). | | | |  |
| **Parameters** | | **Mortality Group**  **(n = 250)** | **Discharged Group**  **(n = 250)** |  |
| AGE | | 0 (0) | 0 (0) |  |
| SEX | | 6 (2.4) | 2 (0.8) |  |
| WEIGHT | | 2 (0.8) | 3 (1.2) |  |
| NO SUBSTANCE ABUSE | | 4 (1.6) | 2 (0.8) |  |
| SMOKING | | 4 (1.6) | 2 (0.8) |  |
| ALCOHOLISM | | 4 (1.6) | 2 (0.8) |  |
| OTHER SUBSTANCE ABUSE | | 4 (1.6) | 2 (0.8) |  |
| GCS-E | | 1 (0.4) | 1 (0.4) |  |
| GCS-V | | 0 (0) | 0 (0) |  |
| GCS-M | | 2 (0.8) | 1 (0.4) |  |
| TEMP | | 7 (2.8) | 4 (1.6) |  |
| HR | | 0 (0) | 1 (0.4) |  |
| BP SYSTOLIC | | 0 (0) | 0 (0) |  |
| BP DIASTOLIC | | 0 (0) | 1 (0.4) |  |
| CLINICAL RR | | 4 (1.6) | 25 (10) |  |
| SpO2 | | 3 (1.2) | 3 (1.2) |  |
| INTUBATED | | 1 (0.4) | 1 (0.4) |  |
| TV | | 44 (17.6) | 106 (42.4) |  |
| RR | | 24 (9.6) | 56 (22.4) |  |
| PEEP | | 37 (14.8) | 96 (38.4) |  |
| FiO2 | | 1 (0.4) | 7 (2.8) |  |
| LACTATE | | 4 (1.6) | 12 (4.8) |  |
| Ph | | 0 (0) | 3 (1.2) |  |
| PCO2 | | 3 (1.2) | 3 (1.2) |  |
| PO2 | | 0 (0) | 4 (1.6) |  |
| HCO3 | | 2 (0.8) | 5 (2) |  |
| BE | | 2 (0.8) | 7 (2.8) |  |
| SUGARS | | 8 (3.2) | 5 (2) |  |
| Ca+ | | 1 (0.4) | 4 (1.6) |  |
| Mg2+ | | 20 (8) | 18 (7.2) |  |
| P/F-Ratio | | 41 (16.4) | 42 (16.8) |  |
| HB | | 1 (0.4) | 0 (0) |  |
| TLC | | 3 (1.2) | 1 (0.4) |  |
| PLT | | 1 (0.4) | 0 (0) |  |
| PT | | 18 (7.2) | 15 (6) |  |
| INR | | 16 (6.4) | 14 (5.6) |  |
| APTT | | 50 (20) | 46 (18.4) |  |
| UREA | | 25 (10) | 22 (8.8) |  |
| CREATININ | | 1 (0.4) | 0 (0) |  |
| Na+ | | 0 (0) | 0 (0) |  |
| k+ | | 0 (0) | 0 (0) |  |
| Cl- | | 37 (14.8) | 30 (12) |  |
| Total-Ca2+ | | 5 (2) | 3 (1.2) |  |
| TB | | 5 (2) | 4 (1.6) |  |
| DB | | 107 (42.8) | 133 (53.2) |  |
| IB | | 0 (0) | 0 (0) |  |
| SGOT | | 2 (0.8) | 2 (0.8) |  |
| SGPT | | 3 (1.2) | 1 (0.4) |  |
| ALP | | 14 (5.6) | 9 (3.6) |  |
| TP | | 0 (0) | 0 (0) |  |
| ALBUMIN | | 14 (5.6) | 4 (1.6) |  |
| PCT | | 43 (17.2) | 55 (22) |  |
| HCT | | 12 (4.8) | 10 (4) |  |
| Phosphate | | 104 (41.6) | 99 (39.6) |  |
| RIGHT LUNG-ZONE1 | | 1 (0.4) | 8 (3.2) |  |
| RIGHT LUNG-ZONE2 | | 1 (0.4) | 7 (2.8) |  |
| RIGHT LUNG-ZONE3 | | 1 (0.4) | 7 (2.8) |  |
| RIGHT LUNG-ZONE4 | | 1 (0.4) | 7 (2.8) |  |
| RIGHT LUNG-ZONE5 | | 0 (0) | 8 (3.2) |  |
| RIGHT LUNG-ZONE6 | | 0 (0) | 9 (3.6) |  |
| LEFT LUNG-ZONE1 | | 3 (1.2) | 6 (2.4) |  |
| LEFT LUNG-ZONE2 | | 2 (0.8) | 6 (2.4) |  |
| LEFT LUNG-ZONE3 | | 2 (0.8) | 7 (2.8) |  |
| LEFT LUNG-ZONE4 | | 1 (0.4) | 6 (2.4) |  |
| LEFT LUNG-ZONE5 | | 1 (0.4) | 6 (2.4) |  |
| LEFT LUNG-ZONE6 | | 1(0.4) | 8 (3.2) |  |
| PLEURAL EFFUSION- R(Y/N) | | 0 (0) | 6 (2.4) |  |
| PLEURAL EFFUSION- L(Y/N) | | 3 (1.2) | 5 (2) |  |
| CONSOLIDATION-R(Y/N) | | 3 (1.2) | 5 (2) |  |
| CONSOLIDATION- L(Y/N) | | 4 (1.6) | 6 (2.4) |  |
| HEART-RA | | 4 (1.6) | 5 (2) |  |
| HEART-RV | | 4 (1.6) | 5 (2) |  |
| HEART-LA | | 5 (2) | 5 (2) |  |
| HEART- LV | | 5 (2) | 5 (2) |  |
| CONTRACTILITY-RV | | 10 (4) | 7 (2.8) |  |
| CONTRACTILITY-LV | | 5 (2) | 6 (2.4) |  |
| LVEF CLASS MARK | | 19 (7.6) | 13 (5.2) |  |
| IVC SIZE | | 45 (18) | 39 (15.6) |  |
| IVC VARIATION | | 34 (13.6) | 22 (8.8) |  |
| DVT Y/N | | 8 (3.2) | 7 (2.8) |  |
| SOFA | | 2 (0.8) | 2 (0.8) |  |
| SOURCE OF SEPSIS | | 1 (0.4) | 0 (0) |  |
| ORGAN DYSFUNCTION | | 1 (0.4) | 0 (0) |  |
| METABOLIC ACID BASE DISORDERS | | 1 (0.4) | 0 (0) |  |
| NUMBER OF VENUS LINES | | 2 (0.8) | 17 (6.8) |  |
| NUMBER OF OTHER LINES/TUBES | | 0 (0) | 4 (1.6) |  |
| NUMBER OF COMORBIDITIES | | 1 (0.4) | 1 (0.4) |  |
| INVASIVE VENTILATION THERAPY | | 0 (0) | 2 (0.8) |  |
| VASOPRESSOR DOSE | | 56 (22.4) | 126 (50.4) |  |
| VASOPRESSOR | | 1 (0.4) | 0 (0) |  |
| STEROIDS | | 2 (0.8) | 3 (1.2) |  |
| ILLNESS TO FIRST ANTIBIOTICS | | 8 (3.2) | 3 (1.2) |  |
| ANTIBIOTICS POST ICU ADMISION | | 1 (0.4) | 0 (0) |  |
| ANTIBIOTICS TILL ICU ADMISSION | | 1 (0.4) | 0 (0) |  |
| DURATION OF ILLNESS BEFORE ICU ADMISSION | | 3 (1.2) | 2 (0.8) |  |
| TYPE OF ICU ADMISSION | | 4 (1.6) | 2 (0.8) |  |
| GCS-Glasgow coma scale; TEMP- Temperature; HR - Heart rate; BP- Blood pressure systolic and diastolic; RR-Respiratory rate; SPO2-Saturation of peripheral oxygen; TV- Tidal volume; PEEP –Positive End Expiratory Pressure; FI02- Fraction of Inspired Oxygen; HB - Hemoglobin; TLC-Total leucocyte count; PLT- Platelet count; PT/INR- Prothrombin time/International normalized ratio; APTT- Activated Partial Thromboplastin Time; Na - Sodium; K+- Potassium; Cl—Chloride; Total ca2+- Total Calcium; TB- Total bilirubin; DB-Direct bilirubin; IB- Indirect Bilirubin; SGOT-Serum Glutamic-oxaloacetic transaminase; SGPT- Serum Glutamate-Pyruvate Transaminase; ALP- Alkaline Phosphatase; TP- Total protein; Alb: Albumin; PCT- Procalcitonin; HCT- Hematocrit; Heart RA- Right Atrium; RV- Right ventricle; LA- Left atrium; LV- Left ventricle; RV- Right ventricle; LVEF- Left ventricular ejection fraction; IVC size- Inferior venacava size; DVT- Deep vein thrombosis; SOFA score- Sequential Organ Failure Assessment score. | | | |  |


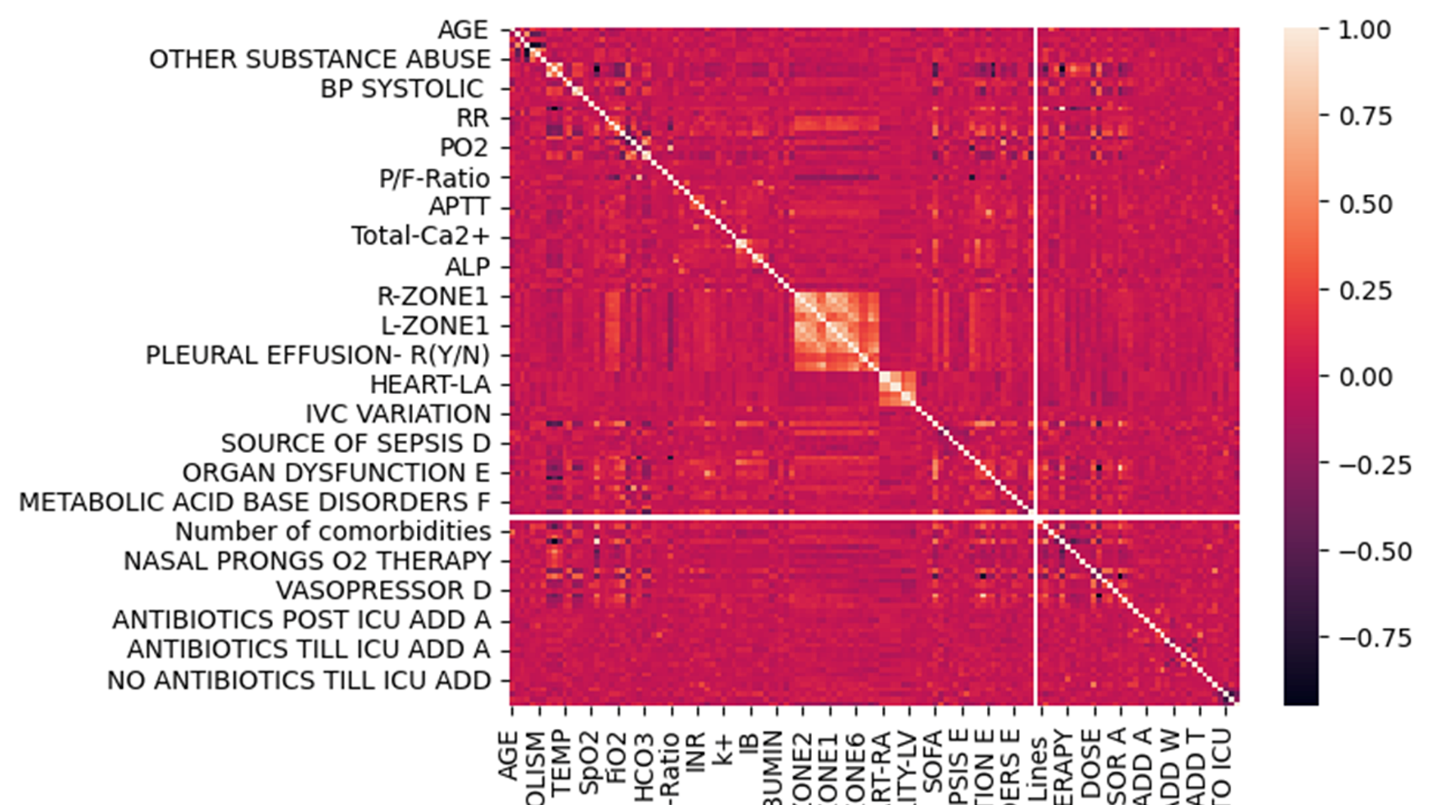


**Supplementary Figure 1:** Pearson correlation plot for demographic and clinical parameters (n=138). The color scheme represents the correlation values in the range +1 to -1. No significant correlations could be observed in the parameters.


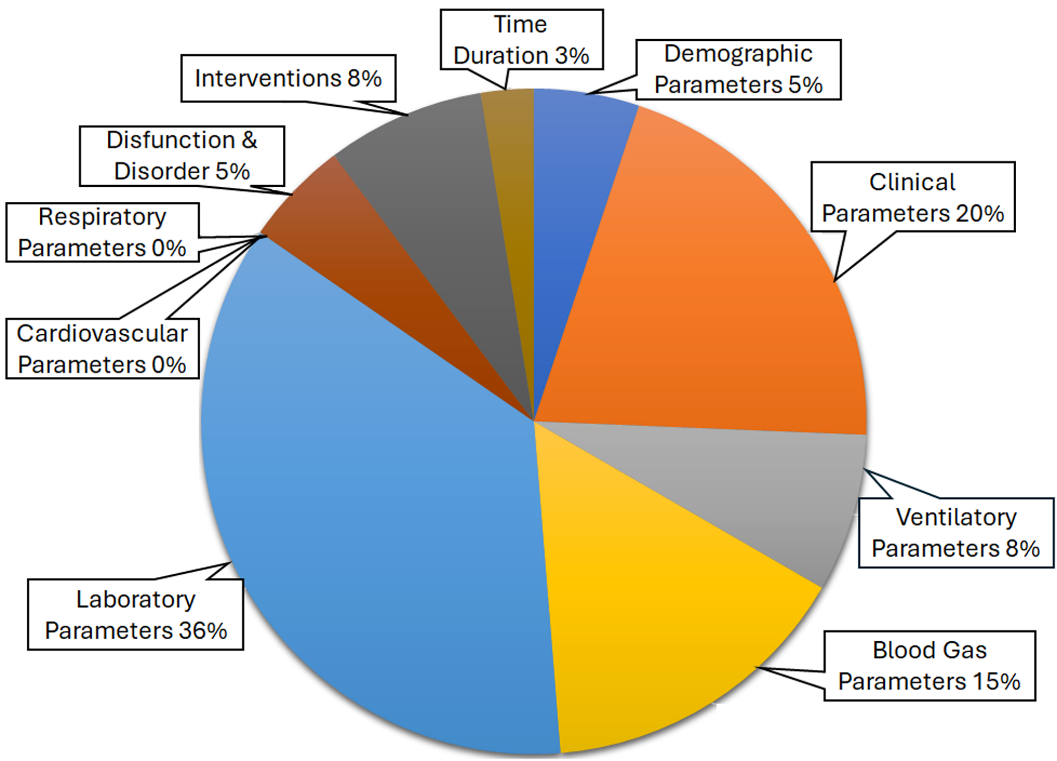


**Supplementary Figure 2:** Distribution of Selected Features (n=39). In the selected top 80% feature set (n=39), Laboratory Parameters displayed the maximum representation (36%), followed by Clinical parameters (20%), Blood Gas parameters (15%), Ventilatory parameters (8%) and Interventions (8%), Dysfunction and Disorder (5%) and Time durations (3%).


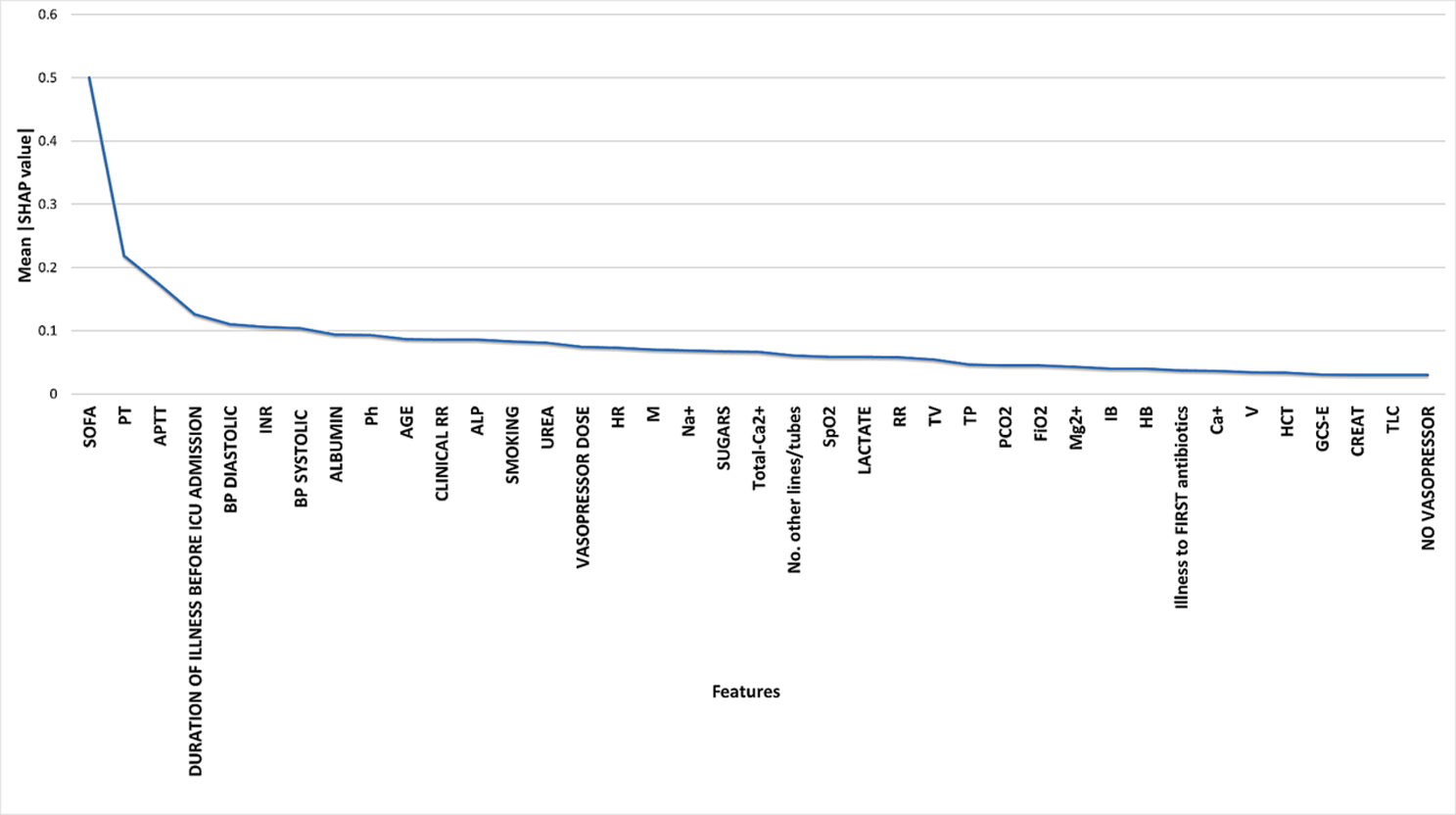


**Supplementary Figure 3:** Selected features N=39 representing 80% of SHAP value spread. The cutoff was set to 20% of the SHAP value spread. The graph above shows a saturation post of these features.


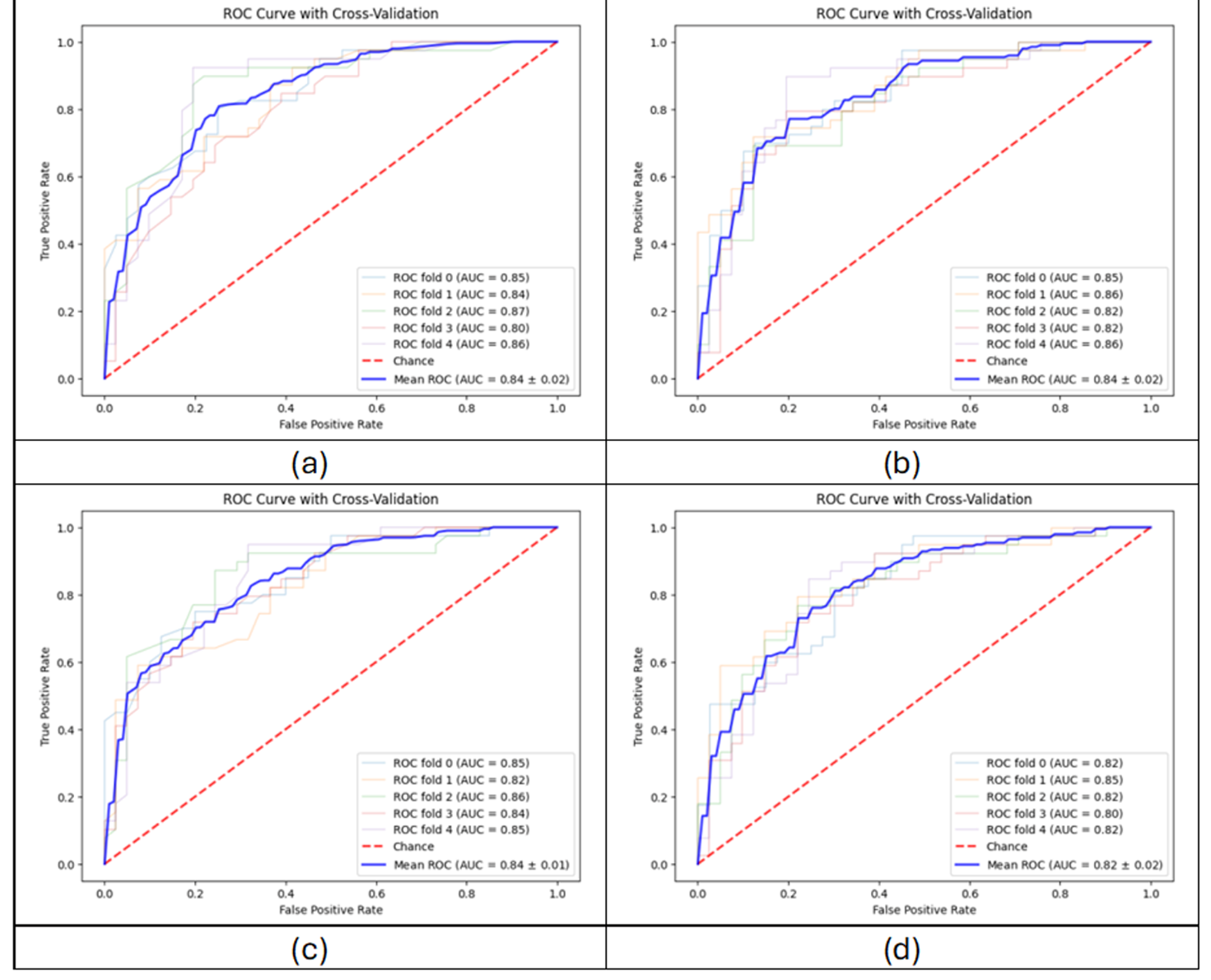


**Supplementary Figure 4:** Plots show AUROC curves for model training using five-fild cross-validation to compare the performance of proposed machine learning models using (a) Random Forest Classifier, (b) XGBoost Classifier, (c) Extra Trees Classifier, and (d) Gradient Boosting Classifier.
